# Crystal structure of azido­(η^5^-cyclo­penta­dien­yl)bis­(tri­phenyl­phosphane-κ*P*)ruthenium(II) di­chloro­methane hemisolvate

**DOI:** 10.1107/S1600536814019187

**Published:** 2014-09-06

**Authors:** Adriana Hernández-Calva, Lidia Meléndez-Balbuena, Maribel Arroyo, Armando Ramírez-Monroy

**Affiliations:** a Centro de Química del Instituto de Ciencias, Benemérita Universidad Autónoma de Puebla, Ciudad Universitaria, San Manuel, 72570, Puebla, Puebla, Mexico; b Facultad de Ciencias Químicas, Benemérita Universidad Autónoma de Puebla, Ciudad Universitaria, San Manuel, 72570, Puebla, Puebla, Mexico

**Keywords:** crystal structure, ruthenium, azido complex, piano-stool geometry

## Abstract

The title solvated complex, [Ru(η^5^-C_5_H_5_)(N_3_){P(C_6_H_5_)_3_}_2_]·0.5CH_2_Cl_2_, displays a typical piano-stool geometry about the Ru^II^ atom. The bond lengths and angles of the cyclo­penta­dienyl and phosphane ligands are very similar to that of the unsolvated complex [Taqui Khan *et al.* (1994 ▶). *Acta Cryst.* C**50**, 502–504]. The azide anion displays similar N—N distances of 1.173 (3) and 1.156 (3) Å and has an N—N—Ru angle of 119.20 (15)°, indicating a greater contribution of the canonical form Ru—N=N^(+)^=N^(-)^ for the bonding situation. An intra­molecular C—H⋯N hydrogen-bonding inter­action between one *ortho* H atom of a phosphane ligand and the N atom coordinating to the metal is observed. A similar inter­molecular inter­action is observed between a *meta* H atom of a phosphane ligand and the terminal azide N atom of a neighbouring complex. Finally, two C—H⋯N inter­actions exists between the H atoms of the di­chloro­methane solvent mol­ecule and the terminal N atom of two azide anions. The solvent mol­ecule is located about a twofold rotation axis and shows disorder of the Cl atoms with an occupancy ratio of 0.62 (3):0.38 (3).

## Related literature   

The structure of the unsolvated ruthenium(II) complex was determined by Taqui Khan *et al.* (1994 ▶). For other azide ruthenium(II) complexes, see: Moura *et al.* (1999 ▶); Govinda­swamy *et al.* (2005 ▶). For metal azide chemistry, see: Fehlhammer & Beck (2013 ▶); Seok & Klapötke (2010 ▶). Non-classical hydrogen bonds were assigned on basis of distances that are shorter than the sum of the van der Waals radii (Bondi, 1964 ▶) of respective atoms. For synthetic details, see: Moura *et al.* (2002 ▶).
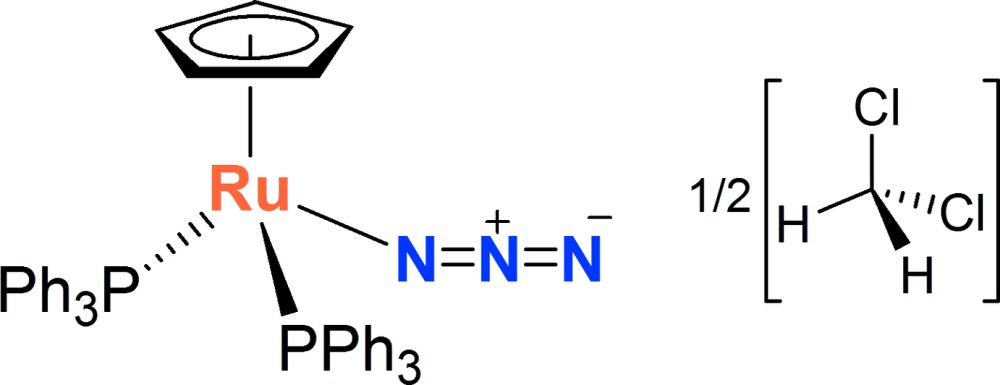


## Experimental   

### Crystal data  

[Ru(C_5_H_5_)(N_3_)(C_18_H_15_P)_2_]·0.5CH_2_Cl_2_
*M* *_r_* = 775.19
Monoclinic, 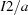

*a* = 20.1817 (4) Å
*b* = 12.4559 (3) Å
*c* = 28.6781 (6) Å
β = 94.213 (2)°
*V* = 7189.7 (3) Å^3^
*Z* = 8
Mo *K*α radiation
μ = 0.63 mm^−1^
*T* = 293 K
0.72 × 0.51 × 0.20 mm

### Data collection  

Agilent Xcalibur Atlas Gemini diffractometer
Absorption correction: analytical [*CrysAlis PRO* (Agilent, 2012 ▶) using a multi=faceted crystal model based on expressions derived by Clark & Reid (1995 ▶)] *T*_min_ = 0.737, *T*_max_ = 0.897
36924 measured reflections
7106 independent reflections
6038 reflections with *I* > 2σ(*I*)
*R*_int_ = 0.032

### Refinement  

*R*[*F* ^2^ > 2σ(*F* ^2^)] = 0.027
*wR*(*F* ^2^) = 0.065
*S* = 1.06
7106 reflections
449 parameters
H-atom parameters constrained
Δρ_max_ = 0.33 e Å^−3^
Δρ_min_ = −0.49 e Å^−3^

### 

Data collection: *CrysAlis PRO* (Agilent, 2012 ▶); cell refinement: *CrysAlis PRO*; data reduction: *CrysAlis RED* (Agilent, 2012 ▶); program(s) used to solve structure: *OLEX2* (Dolomanov *et al.*, 2009 ▶); program(s) used to refine structure: *SHELXL2014* (Sheldrick, 2008 ▶); molecular graphics: *ORTEP-3 for Windows* (Farrugia, 2012 ▶); software used to prepare material for publication: *WinGX* (Farrugia, 2012 ▶).

## Supplementary Material

Crystal structure: contains datablock(s) I, New_Global_Publ_Block. DOI: 10.1107/S1600536814019187/wm5050sup1.cif

Structure factors: contains datablock(s) I. DOI: 10.1107/S1600536814019187/wm5050Isup2.hkl

**Fig. 1.**
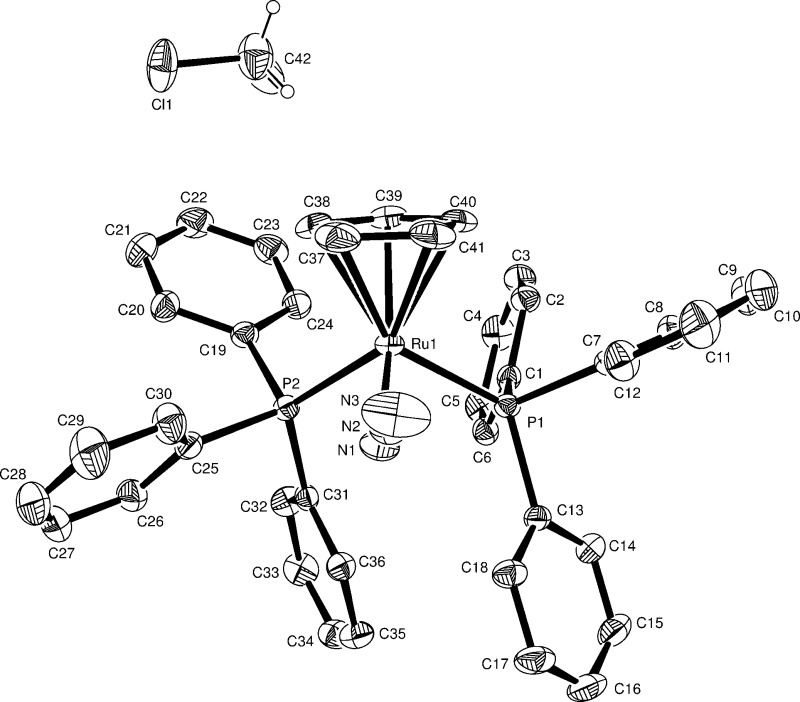
Molecular structure of the title compound showing the atom-numbering scheme. Displacement ellipsoids are drawn at the 30% probability level. Only the major component of the disordered dichloromethane solvate is shown. Hydrogen atoms of the metal complex have been removed for clarity.

. DOI: 10.1107/S1600536814019187/wm5050fig1.tif
Mol­ecular structure of the title compound showing the atom-numbering scheme. Displacement ellipsoids are drawn at the 30% probability level. Only the major component of the disordered di­chloro­methane solvate is shown. Hydrogen atoms of the metal complex have been removed for clarity.

**Fig. 2.**
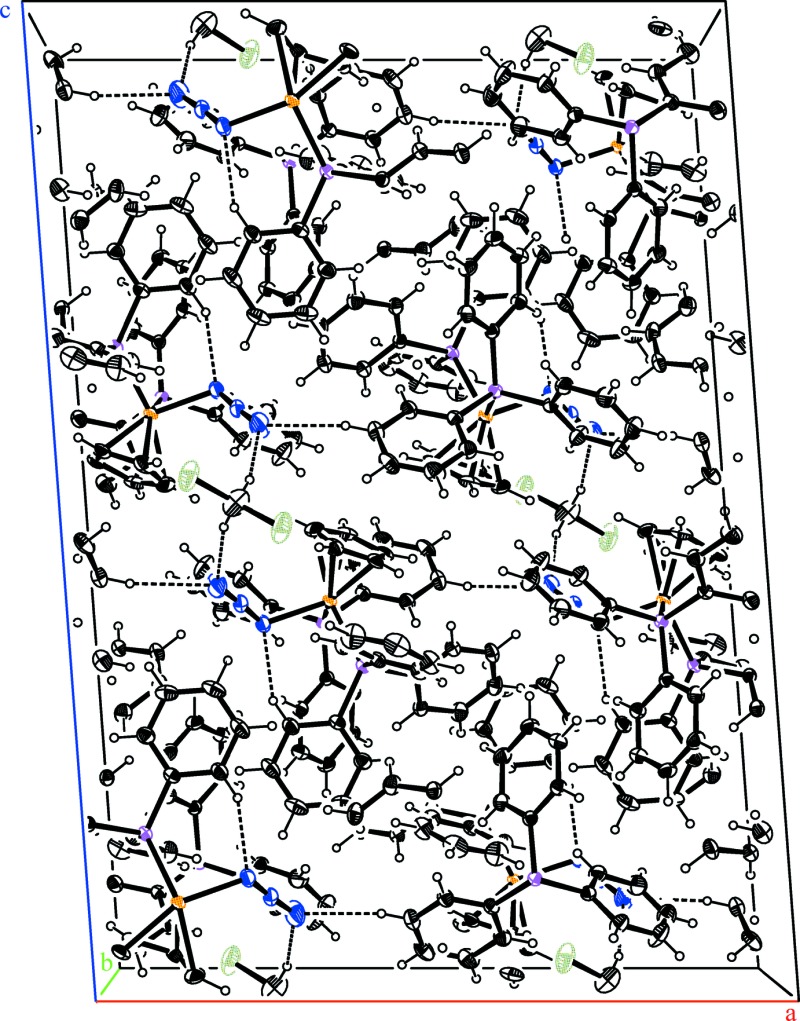
View of the molecular arrangement in the title structure viewed along [010]. Hydrogen bonds are denoted by dashed lines.

. DOI: 10.1107/S1600536814019187/wm5050fig2.tif
View of the mol­ecular arrangement in the title structure viewed along [010]. Hydrogen bonds are denoted by dashed lines.

CCDC reference: 1021189

Additional supporting information:  crystallographic information; 3D view; checkCIF report

## Figures and Tables

**Table 1:** Hydrogen-bond geometry (Å, °)

*D*—H⋯*A*	*D*—H	H⋯*A*	*D*⋯*A*	*D*—H⋯*A*
C18—H18⋯N1	0.93	2.35	3.204 (3)	153
C23—H23⋯N3^i^	0.93	2.62	3.537 (4)	167
C42—H42*A*⋯N3^ii^	0.97	2.4	3.338 (4)	162
C42—H42*B*⋯N3^i^	0.97	2.4	3.338 (4)	162

Symmetry codes: (i) 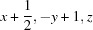; (ii) 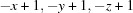.

## supplementary crystallographic information

### S1. Synthesis and crystallization

The title compound was synthesized following a slightly modified procedure developed by Moura *et al.* (2002). Under a dry nitro­gen atmosphere, to [Ru(η5-C_5_H_5_)(PPh_3_)_2_Cl] (0.100 g, 0.138 mmol) dissolved in 20 ml of dry ethanol was added NaN_3_ (0.134 g, 2.061 mmol). The stirred mixture was refluxed for 6 h. After this time, the solvent was removed under vacuum and the product was extracted with 4 ml of dry di­chloro­methane. To the resulting orange solution, 8 ml of degassed hexanes were added in order to induce crystallization at 281 K. Red-orange colored crystals of the title compound were obtained in 82% yield. M.p. 393 K (dec.); IR (KBr) ν(N_3_) 2023 cm^-1^.

### S2. Refinement

All hydrogen atoms were generated at calculated positions with C—H distances constrained to 0.93–0.97 Å. All hydrogen atoms were refined using a riding model approximation with *U*_iso_(H) = 1.2 *U*_eq_(C). The di­chloro­methane molecule is located about a twofold rotation axis. Its chlorine atoms are disordered over two positions, with refined occupancies of 0.62 (3) and 0.38 (3).

### Crystal data

**Table:**

[Ru(C_5_H_5_)(N_3_)(C_18_H_15_P)_2_]·0.5CH_2_Cl_2_	*F*(000) = 3176
*M**_r_* = 775.19	*D*_x_ = 1.432 Mg m^−^^3^
Monoclinic, *I*2/*a*	Mo *K*α radiation, λ = 0.71073 Å
*a* = 20.1817 (4) Å	Cell parameters from 13778 reflections
*b* = 12.4559 (3) Å	θ = 3.5–29.5°
*c* = 28.6781 (6) Å	µ = 0.63 mm^−^^1^
β = 94.213 (2)°	*T* = 293 K
*V* = 7189.7 (3) Å^3^	Prism, orange
*Z* = 8	0.72 × 0.51 × 0.20 mm

### Data collection

**Table:**

Agilent Xcalibur Atlas Gemini diffractometer	7106 independent reflections
Radiation source: Enhance (Mo) X-ray Source	6038 reflections with *I* > 2σ(*I*)
Graphite monochromator	*R*_int_ = 0.032
Detector resolution: 10.5564 pixels mm^-1^	θ_max_ = 26.1°, θ_min_ = 3.3°
ω scans	*h* = −24→24
Absorption correction: analytical [*CrysAlis PRO* (Agilent, 2012) using a multi=faceted crystal model based on expressions derived by Clark & Reid (1995)]	*k* = −15→15
*T*_min_ = 0.737, *T*_max_ = 0.897	*l* = −35→35
36924 measured reflections	

### Refinement

**Table:**

Refinement on *F*^2^	Hydrogen site location: inferred from neighbouring sites
Least-squares matrix: full	H-atom parameters constrained
*R*[*F*^2^ > 2σ(*F*^2^)] = 0.027	*w* = 1/[σ^2^(*F*_o_^2^) + (0.022*P*)^2^ + 9.5067*P*] where *P* = (*F*_o_^2^ + 2*F*_c_^2^)/3
*wR*(*F*^2^) = 0.065	(Δ/σ)_max_ = 0.001
*S* = 1.06	Δρ_max_ = 0.33 e Å^−^^3^
7106 reflections	Δρ_min_ = −0.49 e Å^−^^3^
449 parameters	Extinction correction: *SHELXL*
0 restraints	Extinction coefficient: 0.00036 (4)

### Special details

**Table:**

Experimental. none
Geometry. All e.s.d.'s (except the e.s.d. in the dihedral angle between two l.s. planes) are estimated using the full covariance matrix. The cell e.s.d.'s are taken into account individually in the estimation of e.s.d.'s in distances, angles and torsion angles; correlations between e.s.d.'s in cell parameters are only used when they are defined by crystal symmetry. An approximate (isotropic) treatment of cell e.s.d.'s is used for estimating e.s.d.'s involving l.s. planes.
Refinement. None

### Fractional atomic coordinates and isotropic or equivalent isotropic displacement parameters (Å^2^)

**Table:**

	*x*	*y*	*z*	*U*_iso_*/*U*_eq_	Occ. (<1)
C1	0.49378 (9)	0.46227 (15)	0.32350 (7)	0.0298 (4)	
C2	0.55095 (10)	0.44269 (18)	0.35275 (8)	0.0385 (5)	
H2	0.5499	0.3909	0.3761	0.046*	
C3	0.60895 (11)	0.4986 (2)	0.34772 (9)	0.0471 (6)	
H3	0.6466	0.4837	0.3673	0.057*	
C4	0.61134 (12)	0.5763 (2)	0.31387 (10)	0.0519 (6)	
H4	0.6505	0.6138	0.3104	0.062*	
C5	0.55533 (12)	0.59808 (19)	0.28509 (9)	0.0483 (6)	
H5	0.5567	0.651	0.2623	0.058*	
C6	0.49689 (11)	0.54182 (16)	0.28979 (8)	0.0363 (5)	
H6	0.4594	0.5575	0.2702	0.044*	
C7	0.43752 (10)	0.25218 (16)	0.34102 (7)	0.0344 (5)	
C8	0.49774 (12)	0.20901 (19)	0.33053 (9)	0.0461 (6)	
H8	0.5292	0.2527	0.3178	0.055*	
C9	0.51178 (15)	0.1019 (2)	0.33864 (11)	0.0608 (7)	
H9	0.5529	0.0743	0.332	0.073*	
C10	0.46576 (18)	0.0364 (2)	0.35632 (11)	0.0688 (9)	
H10	0.4758	−0.0353	0.3625	0.083*	
C11	0.40434 (17)	0.0766 (2)	0.36496 (12)	0.0711 (9)	
H11	0.3722	0.0313	0.3758	0.085*	
C12	0.39027 (13)	0.18415 (19)	0.35761 (10)	0.0525 (6)	
H12	0.3488	0.211	0.3638	0.063*	
C13	0.37121 (10)	0.38670 (16)	0.27572 (7)	0.0320 (4)	
C14	0.40484 (12)	0.37479 (18)	0.23548 (8)	0.0431 (5)	
H14	0.451	0.374	0.2376	0.052*	
C15	0.37038 (14)	0.3640 (2)	0.19232 (9)	0.0540 (7)	
H15	0.3935	0.3564	0.1656	0.065*	
C16	0.30208 (15)	0.3646 (2)	0.18854 (9)	0.0582 (7)	
H16	0.279	0.3588	0.1594	0.07*	
C17	0.26818 (13)	0.3737 (2)	0.22806 (9)	0.0554 (7)	
H17	0.222	0.3728	0.2256	0.066*	
C18	0.30229 (11)	0.38420 (18)	0.27166 (8)	0.0422 (5)	
H18	0.2788	0.3896	0.2983	0.051*	
C19	0.42371 (10)	0.72523 (17)	0.41116 (7)	0.0350 (5)	
C20	0.40982 (12)	0.79993 (19)	0.44478 (8)	0.0454 (6)	
H20	0.366	0.8123	0.4514	0.054*	
C21	0.46075 (14)	0.8563 (2)	0.46864 (10)	0.0589 (7)	
H21	0.4509	0.9064	0.4912	0.071*	
C22	0.52559 (14)	0.8392 (2)	0.45935 (10)	0.0585 (7)	
H22	0.5595	0.8771	0.4757	0.07*	
C23	0.54049 (12)	0.7659 (2)	0.42584 (9)	0.0516 (6)	
H23	0.5844	0.7547	0.4192	0.062*	
C24	0.48992 (11)	0.70906 (19)	0.40198 (8)	0.0433 (5)	
H24	0.5002	0.6592	0.3795	0.052*	
C25	0.28225 (10)	0.70450 (18)	0.39722 (7)	0.0360 (5)	
C26	0.26325 (12)	0.8048 (2)	0.37980 (9)	0.0479 (6)	
H26	0.2895	0.8397	0.3592	0.058*	
C27	0.20570 (14)	0.8534 (2)	0.39266 (10)	0.0627 (8)	
H27	0.1937	0.9208	0.3809	0.075*	
C28	0.16642 (15)	0.8026 (3)	0.42271 (11)	0.0722 (9)	
H28	0.1277	0.8351	0.4314	0.087*	
C29	0.18428 (16)	0.7042 (3)	0.43981 (12)	0.0785 (10)	
H29	0.1575	0.6696	0.4601	0.094*	
C30	0.24230 (13)	0.6544 (2)	0.42730 (9)	0.0551 (7)	
H30	0.254	0.5872	0.4394	0.066*	
C31	0.35924 (10)	0.68670 (16)	0.32038 (7)	0.0307 (4)	
C32	0.39666 (11)	0.77203 (19)	0.30534 (8)	0.0438 (5)	
H32	0.4261	0.8076	0.3265	0.053*	
C33	0.39017 (12)	0.8041 (2)	0.25902 (9)	0.0498 (6)	
H33	0.4145	0.8625	0.2494	0.06*	
C34	0.34825 (12)	0.7507 (2)	0.22735 (8)	0.0493 (6)	
H34	0.3449	0.7716	0.1961	0.059*	
C35	0.31111 (12)	0.6662 (2)	0.24174 (8)	0.0471 (6)	
H35	0.2828	0.6295	0.2202	0.057*	
C36	0.31569 (11)	0.63574 (17)	0.28805 (7)	0.0368 (5)	
H36	0.2891	0.5802	0.2977	0.044*	
C37	0.35806 (14)	0.4319 (3)	0.47670 (9)	0.0638 (8)	
H37	0.3159	0.4377	0.4914	0.077*	
C38	0.40514 (15)	0.5138 (2)	0.47330 (8)	0.0561 (7)	
H38	0.4015	0.5871	0.4854	0.067*	
C39	0.45983 (12)	0.4716 (2)	0.45186 (8)	0.0482 (6)	
H39	0.5012	0.5096	0.4469	0.058*	
C40	0.44716 (12)	0.3625 (2)	0.44317 (8)	0.0478 (6)	
H40	0.478	0.3118	0.4301	0.057*	
C41	0.38453 (14)	0.3368 (2)	0.45750 (9)	0.0576 (7)	
H41	0.3639	0.2655	0.4566	0.069*	
C42	0.75	0.8824 (4)	0.5	0.0924 (16)	
H42A	0.7713	0.8364	0.5239	0.111*	0.5
H42B	0.7287	0.8364	0.4761	0.111*	0.5
N1	0.27184 (9)	0.42428 (17)	0.37835 (7)	0.0448 (5)	
N2	0.24204 (9)	0.36167 (17)	0.39907 (7)	0.0442 (5)	
N3	0.20984 (13)	0.3008 (3)	0.41775 (11)	0.0928 (10)	
P1	0.41536 (2)	0.39566 (4)	0.33427 (2)	0.02831 (12)	
P2	0.35969 (3)	0.64007 (4)	0.38088 (2)	0.03007 (12)	
Ru1	0.37307 (2)	0.46072 (2)	0.40230 (2)	0.02988 (7)	
Cl1	0.6866 (3)	0.9604 (3)	0.52581 (15)	0.095 (2)	0.62 (3)
Cl1A	0.7012 (8)	0.9520 (10)	0.5236 (4)	0.202 (7)	0.38 (3)

### Atomic displacement parameters (Å^2^)

**Table:**

	*U*^11^	*U*^22^	*U*^33^	*U*^12^	*U*^13^	*U*^23^
C1	0.0275 (10)	0.0285 (10)	0.0335 (11)	−0.0023 (8)	0.0018 (8)	−0.0054 (8)
C2	0.0348 (11)	0.0401 (12)	0.0399 (12)	−0.0015 (9)	−0.0021 (9)	−0.0012 (10)
C3	0.0309 (11)	0.0516 (14)	0.0573 (15)	−0.0033 (11)	−0.0067 (10)	−0.0057 (12)
C4	0.0354 (13)	0.0510 (15)	0.0700 (18)	−0.0136 (11)	0.0088 (12)	−0.0033 (13)
C5	0.0481 (14)	0.0410 (13)	0.0563 (15)	−0.0072 (11)	0.0080 (12)	0.0065 (11)
C6	0.0329 (11)	0.0338 (11)	0.0421 (12)	0.0003 (9)	0.0019 (9)	−0.0028 (9)
C7	0.0378 (11)	0.0292 (11)	0.0349 (11)	−0.0014 (9)	−0.0065 (9)	−0.0007 (9)
C8	0.0497 (14)	0.0359 (12)	0.0526 (15)	0.0029 (11)	0.0023 (11)	−0.0033 (11)
C9	0.0625 (17)	0.0407 (15)	0.077 (2)	0.0136 (13)	−0.0066 (15)	−0.0112 (14)
C10	0.096 (2)	0.0297 (13)	0.078 (2)	0.0058 (15)	−0.0107 (18)	−0.0013 (13)
C11	0.087 (2)	0.0357 (14)	0.091 (2)	−0.0197 (15)	0.0089 (18)	0.0075 (15)
C12	0.0510 (14)	0.0377 (13)	0.0691 (18)	−0.0081 (11)	0.0071 (13)	0.0015 (12)
C13	0.0382 (11)	0.0249 (10)	0.0318 (11)	−0.0031 (9)	−0.0052 (9)	0.0007 (8)
C14	0.0462 (13)	0.0418 (13)	0.0406 (13)	−0.0009 (10)	−0.0016 (10)	−0.0065 (10)
C15	0.0780 (19)	0.0496 (15)	0.0337 (13)	−0.0074 (13)	−0.0002 (12)	−0.0066 (11)
C16	0.0781 (19)	0.0524 (15)	0.0404 (14)	−0.0139 (14)	−0.0213 (13)	−0.0007 (12)
C17	0.0486 (14)	0.0575 (16)	0.0562 (17)	−0.0130 (12)	−0.0213 (12)	0.0006 (13)
C18	0.0398 (12)	0.0444 (13)	0.0411 (13)	−0.0081 (10)	−0.0060 (10)	0.0011 (10)
C19	0.0355 (11)	0.0340 (11)	0.0347 (11)	−0.0022 (9)	−0.0020 (9)	−0.0014 (9)
C20	0.0449 (13)	0.0444 (13)	0.0463 (14)	−0.0006 (11)	−0.0002 (11)	−0.0106 (11)
C21	0.0678 (18)	0.0505 (16)	0.0567 (17)	−0.0048 (13)	−0.0071 (14)	−0.0197 (13)
C22	0.0559 (16)	0.0509 (15)	0.0648 (18)	−0.0131 (13)	−0.0214 (13)	−0.0070 (13)
C23	0.0366 (13)	0.0532 (15)	0.0634 (17)	−0.0039 (11)	−0.0069 (11)	0.0034 (13)
C24	0.0390 (12)	0.0425 (13)	0.0477 (14)	0.0000 (10)	−0.0021 (10)	−0.0055 (11)
C25	0.0340 (11)	0.0439 (13)	0.0300 (11)	0.0017 (10)	0.0020 (9)	−0.0062 (9)
C26	0.0478 (14)	0.0539 (15)	0.0425 (13)	0.0109 (12)	0.0062 (11)	0.0011 (11)
C27	0.0627 (17)	0.0670 (18)	0.0582 (17)	0.0276 (15)	0.0039 (14)	−0.0034 (14)
C28	0.0520 (17)	0.094 (2)	0.072 (2)	0.0228 (17)	0.0162 (15)	−0.0151 (18)
C29	0.0668 (19)	0.091 (2)	0.083 (2)	0.0081 (18)	0.0440 (18)	0.0031 (19)
C30	0.0544 (15)	0.0570 (16)	0.0567 (16)	0.0065 (13)	0.0219 (13)	0.0033 (13)
C31	0.0305 (10)	0.0302 (10)	0.0318 (11)	0.0047 (8)	0.0045 (8)	−0.0003 (8)
C32	0.0426 (13)	0.0443 (13)	0.0443 (13)	−0.0107 (11)	0.0028 (10)	0.0006 (11)
C33	0.0496 (14)	0.0524 (15)	0.0489 (15)	−0.0078 (12)	0.0133 (12)	0.0144 (12)
C34	0.0499 (14)	0.0645 (16)	0.0341 (12)	0.0049 (13)	0.0071 (11)	0.0131 (12)
C35	0.0494 (14)	0.0566 (15)	0.0344 (12)	−0.0012 (12)	−0.0036 (10)	0.0010 (11)
C36	0.0389 (12)	0.0350 (11)	0.0363 (12)	−0.0018 (9)	0.0023 (9)	0.0016 (9)
C37	0.0580 (16)	0.102 (2)	0.0320 (13)	0.0178 (17)	0.0087 (12)	0.0233 (14)
C38	0.0784 (19)	0.0633 (17)	0.0244 (12)	0.0088 (15)	−0.0119 (12)	−0.0029 (11)
C39	0.0466 (14)	0.0661 (17)	0.0294 (12)	−0.0040 (12)	−0.0137 (10)	0.0038 (11)
C40	0.0513 (14)	0.0582 (16)	0.0318 (12)	0.0129 (12)	−0.0117 (10)	0.0097 (11)
C41	0.0657 (17)	0.0597 (17)	0.0453 (15)	−0.0064 (14)	−0.0110 (13)	0.0264 (13)
C42	0.107 (4)	0.071 (3)	0.103 (4)	0	0.038 (3)	0
N1	0.0325 (10)	0.0552 (12)	0.0459 (11)	−0.0045 (9)	−0.0029 (9)	0.0100 (10)
N2	0.0315 (10)	0.0549 (13)	0.0459 (12)	−0.0017 (9)	0.0014 (9)	0.0036 (10)
N3	0.0596 (16)	0.122 (2)	0.095 (2)	−0.0356 (17)	−0.0020 (15)	0.0483 (19)
P1	0.0268 (3)	0.0274 (3)	0.0300 (3)	−0.0018 (2)	−0.0031 (2)	−0.0001 (2)
P2	0.0296 (3)	0.0323 (3)	0.0282 (3)	−0.0013 (2)	0.0011 (2)	−0.0018 (2)
Ru1	0.02908 (10)	0.03461 (10)	0.02536 (10)	−0.00068 (7)	−0.00197 (6)	0.00396 (7)
Cl1	0.0622 (18)	0.091 (2)	0.137 (3)	−0.0075 (10)	0.0361 (19)	−0.0484 (14)
Cl1A	0.130 (6)	0.165 (6)	0.331 (15)	−0.006 (4)	0.148 (9)	−0.068 (5)

### Geometric parameters (Å, º)

**Table:**

C1—C6	1.389 (3)	C25—P2	1.848 (2)
C1—C2	1.397 (3)	C26—C27	1.384 (3)
C1—P1	1.833 (2)	C26—H26	0.93
C2—C3	1.379 (3)	C27—C28	1.368 (4)
C2—H2	0.93	C27—H27	0.93
C3—C4	1.374 (4)	C28—C29	1.359 (4)
C3—H3	0.93	C28—H28	0.93
C4—C5	1.377 (3)	C29—C30	1.395 (4)
C4—H4	0.93	C29—H29	0.93
C5—C6	1.387 (3)	C30—H30	0.93
C5—H5	0.93	C31—C36	1.384 (3)
C6—H6	0.93	C31—C32	1.391 (3)
C7—C8	1.382 (3)	C31—P2	1.829 (2)
C7—C12	1.386 (3)	C32—C33	1.384 (3)
C7—P1	1.849 (2)	C32—H32	0.93
C8—C9	1.380 (3)	C33—C34	1.368 (3)
C8—H8	0.93	C33—H33	0.93
C9—C10	1.362 (4)	C34—C35	1.373 (3)
C9—H9	0.93	C34—H34	0.93
C10—C11	1.376 (4)	C35—C36	1.378 (3)
C10—H10	0.93	C35—H35	0.93
C11—C12	1.382 (4)	C36—H36	0.93
C11—H11	0.93	C37—C38	1.403 (4)
C12—H12	0.93	C37—C41	1.426 (4)
C13—C18	1.388 (3)	C37—Ru1	2.207 (2)
C13—C14	1.389 (3)	C37—H37	0.98
C13—P1	1.846 (2)	C38—C39	1.404 (4)
C14—C15	1.381 (3)	C38—Ru1	2.193 (2)
C14—H14	0.93	C38—H38	0.98
C15—C16	1.375 (4)	C39—C40	1.402 (4)
C15—H15	0.93	C39—Ru1	2.177 (2)
C16—C17	1.371 (4)	C39—H39	0.98
C16—H16	0.93	C40—C41	1.395 (4)
C17—C18	1.389 (3)	C40—Ru1	2.202 (2)
C17—H17	0.93	C40—H40	0.98
C18—H18	0.93	C41—Ru1	2.211 (2)
C19—C20	1.383 (3)	C41—H41	0.98
C19—C24	1.395 (3)	C42—Cl1A^i^	1.508 (16)
C19—P2	1.840 (2)	C42—Cl1A	1.508 (16)
C20—C21	1.384 (3)	C42—Cl1^i^	1.807 (7)
C20—H20	0.93	C42—Cl1	1.807 (7)
C21—C22	1.371 (4)	C42—H42A	0.97
C21—H21	0.93	C42—H42B	0.97
C22—C23	1.374 (4)	N1—N2	1.173 (3)
C22—H22	0.93	N1—Ru1	2.1553 (18)
C23—C24	1.382 (3)	N2—N3	1.156 (3)
C23—H23	0.93	P1—Ru1	2.3314 (6)
C24—H24	0.93	P2—Ru1	2.3274 (6)
C25—C30	1.373 (3)	Cl1A—Cl1A^i^	2.47 (3)
C25—C26	1.389 (3)		
			
C6—C1—C2	117.70 (19)	C33—C32—H32	119.9
C6—C1—P1	121.59 (15)	C31—C32—H32	119.9
C2—C1—P1	120.33 (16)	C34—C33—C32	120.5 (2)
C3—C2—C1	121.3 (2)	C34—C33—H33	119.8
C3—C2—H2	119.4	C32—C33—H33	119.8
C1—C2—H2	119.4	C33—C34—C35	119.9 (2)
C4—C3—C2	120.3 (2)	C33—C34—H34	120.1
C4—C3—H3	119.9	C35—C34—H34	120.1
C2—C3—H3	119.9	C34—C35—C36	120.1 (2)
C3—C4—C5	119.5 (2)	C34—C35—H35	120
C3—C4—H4	120.2	C36—C35—H35	120
C5—C4—H4	120.2	C35—C36—C31	120.9 (2)
C4—C5—C6	120.6 (2)	C35—C36—H36	119.5
C4—C5—H5	119.7	C31—C36—H36	119.5
C6—C5—H5	119.7	C38—C37—C41	107.6 (2)
C5—C6—C1	120.7 (2)	C38—C37—Ru1	70.89 (14)
C5—C6—H6	119.7	C41—C37—Ru1	71.33 (14)
C1—C6—H6	119.7	C38—C37—H37	126.1
C8—C7—C12	118.4 (2)	C41—C37—H37	126.1
C8—C7—P1	124.26 (17)	Ru1—C37—H37	126.1
C12—C7—P1	117.37 (17)	C37—C38—C39	108.4 (3)
C9—C8—C7	120.8 (2)	C37—C38—Ru1	71.92 (15)
C9—C8—H8	119.6	C39—C38—Ru1	70.62 (13)
C7—C8—H8	119.6	C37—C38—H38	125.7
C10—C9—C8	120.3 (3)	C39—C38—H38	125.7
C10—C9—H9	119.8	Ru1—C38—H38	125.7
C8—C9—H9	119.8	C40—C39—C38	107.6 (2)
C9—C10—C11	119.8 (3)	C40—C39—Ru1	72.30 (13)
C9—C10—H10	120.1	C38—C39—Ru1	71.88 (13)
C11—C10—H10	120.1	C40—C39—H39	126.1
C10—C11—C12	120.2 (3)	C38—C39—H39	126.1
C10—C11—H11	119.9	Ru1—C39—H39	126.1
C12—C11—H11	119.9	C41—C40—C39	109.1 (2)
C11—C12—C7	120.4 (3)	C41—C40—Ru1	71.92 (13)
C11—C12—H12	119.8	C39—C40—Ru1	70.34 (13)
C7—C12—H12	119.8	C41—C40—H40	125.4
C18—C13—C14	118.4 (2)	C39—C40—H40	125.4
C18—C13—P1	119.45 (17)	Ru1—C40—H40	125.4
C14—C13—P1	121.98 (16)	C40—C41—C37	107.3 (3)
C15—C14—C13	120.7 (2)	C40—C41—Ru1	71.24 (13)
C15—C14—H14	119.7	C37—C41—Ru1	71.00 (14)
C13—C14—H14	119.7	C40—C41—H41	126.3
C16—C15—C14	120.4 (2)	C37—C41—H41	126.3
C16—C15—H15	119.8	Ru1—C41—H41	126.3
C14—C15—H15	119.8	Cl1A^i^—C42—Cl1A	109.8 (9)
C17—C16—C15	119.6 (2)	Cl1^i^—C42—Cl1	114.9 (4)
C17—C16—H16	120.2	Cl1^i^—C42—H42A	108.5
C15—C16—H16	120.2	Cl1—C42—H42A	108.5
C16—C17—C18	120.5 (2)	Cl1^i^—C42—H42B	108.5
C16—C17—H17	119.7	Cl1—C42—H42B	108.5
C18—C17—H17	119.7	H42A—C42—H42B	107.5
C13—C18—C17	120.3 (2)	N2—N1—Ru1	119.20 (15)
C13—C18—H18	119.8	N3—N2—N1	176.3 (3)
C17—C18—H18	119.8	C1—P1—C13	103.84 (9)
C20—C19—C24	118.3 (2)	C1—P1—C7	104.47 (9)
C20—C19—P2	123.07 (17)	C13—P1—C7	97.68 (9)
C24—C19—P2	118.47 (16)	C1—P1—Ru1	110.94 (6)
C19—C20—C21	120.3 (2)	C13—P1—Ru1	126.90 (7)
C19—C20—H20	119.8	C7—P1—Ru1	110.42 (7)
C21—C20—H20	119.8	C31—P2—C19	102.66 (10)
C22—C21—C20	120.6 (2)	C31—P2—C25	99.05 (9)
C22—C21—H21	119.7	C19—P2—C25	101.98 (10)
C20—C21—H21	119.7	C31—P2—Ru1	123.20 (7)
C21—C22—C23	120.0 (2)	C19—P2—Ru1	111.41 (7)
C21—C22—H22	120	C25—P2—Ru1	115.71 (7)
C23—C22—H22	120	N1—Ru1—C39	156.83 (9)
C22—C23—C24	119.7 (2)	N1—Ru1—C38	124.58 (10)
C22—C23—H23	120.2	C39—Ru1—C38	37.49 (10)
C24—C23—H23	120.2	N1—Ru1—C40	130.04 (9)
C23—C24—C19	121.0 (2)	C39—Ru1—C40	37.35 (9)
C23—C24—H24	119.5	C38—Ru1—C40	62.02 (10)
C19—C24—H24	119.5	N1—Ru1—C37	94.57 (10)
C30—C25—C26	118.4 (2)	C39—Ru1—C37	62.60 (10)
C30—C25—P2	120.58 (18)	C38—Ru1—C37	37.19 (11)
C26—C25—P2	121.05 (17)	C40—Ru1—C37	62.03 (10)
C27—C26—C25	120.8 (2)	N1—Ru1—C41	97.36 (9)
C27—C26—H26	119.6	C39—Ru1—C41	62.57 (10)
C25—C26—H26	119.6	C38—Ru1—C41	62.45 (11)
C28—C27—C26	120.1 (3)	C40—Ru1—C41	36.85 (9)
C28—C27—H27	119.9	C37—Ru1—C41	37.67 (11)
C26—C27—H27	119.9	N1—Ru1—P2	91.67 (6)
C29—C28—C27	119.6 (3)	C39—Ru1—P2	100.63 (7)
C29—C28—H28	120.2	C38—Ru1—P2	88.63 (8)
C27—C28—H28	120.2	C40—Ru1—P2	137.30 (7)
C28—C29—C30	120.9 (3)	C37—Ru1—P2	112.95 (9)
C28—C29—H29	119.6	C41—Ru1—P2	149.66 (8)
C30—C29—H29	119.6	N1—Ru1—P1	93.26 (6)
C25—C30—C29	120.2 (3)	C39—Ru1—P1	103.99 (7)
C25—C30—H30	119.9	C38—Ru1—P1	141.32 (8)
C29—C30—H30	119.9	C40—Ru1—P1	88.64 (7)
C36—C31—C32	118.4 (2)	C37—Ru1—P1	146.86 (8)
C36—C31—P2	116.65 (16)	C41—Ru1—P1	109.34 (8)
C32—C31—P2	124.88 (16)	P2—Ru1—P1	98.933 (19)
C33—C32—C31	120.2 (2)	C42—Cl1A—Cl1A^i^	35.1 (4)
			
C6—C1—C2—C3	−1.4 (3)	C37—C38—C39—C40	−1.6 (3)
P1—C1—C2—C3	−174.44 (18)	Ru1—C38—C39—C40	−63.96 (15)
C1—C2—C3—C4	0.8 (4)	C37—C38—C39—Ru1	62.36 (17)
C2—C3—C4—C5	0.2 (4)	C38—C39—C40—C41	1.9 (3)
C3—C4—C5—C6	−0.5 (4)	Ru1—C39—C40—C41	−61.81 (16)
C4—C5—C6—C1	−0.2 (4)	C38—C39—C40—Ru1	63.68 (15)
C2—C1—C6—C5	1.1 (3)	C39—C40—C41—C37	−1.4 (3)
P1—C1—C6—C5	174.04 (17)	Ru1—C40—C41—C37	−62.25 (16)
C12—C7—C8—C9	−3.3 (4)	C39—C40—C41—Ru1	60.82 (16)
P1—C7—C8—C9	176.9 (2)	C38—C37—C41—C40	0.4 (3)
C7—C8—C9—C10	1.3 (4)	Ru1—C37—C41—C40	62.40 (16)
C8—C9—C10—C11	1.7 (5)	C38—C37—C41—Ru1	−61.98 (17)
C9—C10—C11—C12	−2.7 (5)	C6—C1—P1—C13	35.67 (19)
C10—C11—C12—C7	0.7 (5)	C2—C1—P1—C13	−151.58 (17)
C8—C7—C12—C11	2.3 (4)	C6—C1—P1—C7	137.55 (17)
P1—C7—C12—C11	−177.9 (2)	C2—C1—P1—C7	−49.71 (19)
C18—C13—C14—C15	2.0 (3)	C6—C1—P1—Ru1	−103.47 (17)
P1—C13—C14—C15	177.42 (18)	C2—C1—P1—Ru1	69.28 (18)
C13—C14—C15—C16	−0.2 (4)	C18—C13—P1—C1	−152.65 (17)
C14—C15—C16—C17	−1.3 (4)	C14—C13—P1—C1	31.9 (2)
C15—C16—C17—C18	1.1 (4)	C18—C13—P1—C7	100.32 (18)
C14—C13—C18—C17	−2.1 (3)	C14—C13—P1—C7	−75.09 (19)
P1—C13—C18—C17	−177.72 (18)	C18—C13—P1—Ru1	−22.5 (2)
C16—C17—C18—C13	0.6 (4)	C14—C13—P1—Ru1	162.12 (15)
C24—C19—C20—C21	−0.3 (4)	C8—C7—P1—C1	−11.1 (2)
P2—C19—C20—C21	175.5 (2)	C12—C7—P1—C1	169.00 (18)
C19—C20—C21—C22	0.0 (4)	C8—C7—P1—C13	95.4 (2)
C20—C21—C22—C23	0.4 (4)	C12—C7—P1—C13	−84.49 (19)
C21—C22—C23—C24	−0.7 (4)	C8—C7—P1—Ru1	−130.47 (18)
C22—C23—C24—C19	0.4 (4)	C12—C7—P1—Ru1	49.67 (19)
C20—C19—C24—C23	0.1 (3)	C36—C31—P2—C19	179.14 (16)
P2—C19—C24—C23	−175.91 (19)	C32—C31—P2—C19	−4.2 (2)
C30—C25—C26—C27	0.4 (4)	C36—C31—P2—C25	−76.31 (17)
P2—C25—C26—C27	−179.1 (2)	C32—C31—P2—C25	100.35 (19)
C25—C26—C27—C28	−0.4 (4)	C36—C31—P2—Ru1	52.69 (18)
C26—C27—C28—C29	0.0 (5)	C32—C31—P2—Ru1	−130.65 (17)
C27—C28—C29—C30	0.3 (5)	C20—C19—P2—C31	114.6 (2)
C26—C25—C30—C29	−0.1 (4)	C24—C19—P2—C31	−69.56 (19)
P2—C25—C30—C29	179.4 (2)	C20—C19—P2—C25	12.4 (2)
C28—C29—C30—C25	−0.2 (5)	C24—C19—P2—C25	−171.83 (18)
C36—C31—C32—C33	−0.2 (3)	C20—C19—P2—Ru1	−111.66 (19)
P2—C31—C32—C33	−176.82 (18)	C24—C19—P2—Ru1	64.14 (19)
C31—C32—C33—C34	−1.7 (4)	C30—C25—P2—C31	145.2 (2)
C32—C33—C34—C35	1.6 (4)	C26—C25—P2—C31	−35.4 (2)
C33—C34—C35—C36	0.5 (4)	C30—C25—P2—C19	−109.7 (2)
C34—C35—C36—C31	−2.4 (4)	C26—C25—P2—C19	69.8 (2)
C32—C31—C36—C35	2.2 (3)	C30—C25—P2—Ru1	11.4 (2)
P2—C31—C36—C35	179.13 (18)	C26—C25—P2—Ru1	−169.16 (16)
C41—C37—C38—C39	0.7 (3)	Cl1^i^—C42—Cl1A—Cl1A^i^	3.8 (7)
Ru1—C37—C38—C39	−61.54 (16)	Cl1—C42—Cl1A—Cl1A^i^	−126 (9)
C41—C37—C38—Ru1	62.26 (17)		

Symmetry code: (i) −*x*+3/2, *y*, −*z*+1.

### Hydrogen-bond geometry (Å, º)

**Table:**

*D*—H···*A*	*D*—H	H···*A*	*D*···*A*	*D*—H···*A*
C18—H18···N1	0.93	2.35	3.204 (3)	153
C23—H23···N3^ii^	0.93	2.62	3.537 (4)	167
C42—H42*A*···N3^iii^	0.97	2.4	3.338 (4)	162
C42—H42*B*···N3^ii^	0.97	2.4	3.338 (4)	162

Symmetry codes: (ii) *x*+1/2, −*y*+1, *z*; (iii) −*x*+1, −*y*+1, −*z*+1.
