# Supplementary figures and images for: Crystal structure of azido­(η5-cyclo­penta­dien­yl)bis­(tri­phenyl­phosphane-κP)ruthenium(II) di­chloro­methane hemisolvate
Source: Acta Crystallogr Sect E Struct Rep Online. 2014 Sep 6;70(Pt 10):m345–6. doi: 10.1107/S1600536814019187 (PMC4257169; doi:10.1107/S1600536814019187)

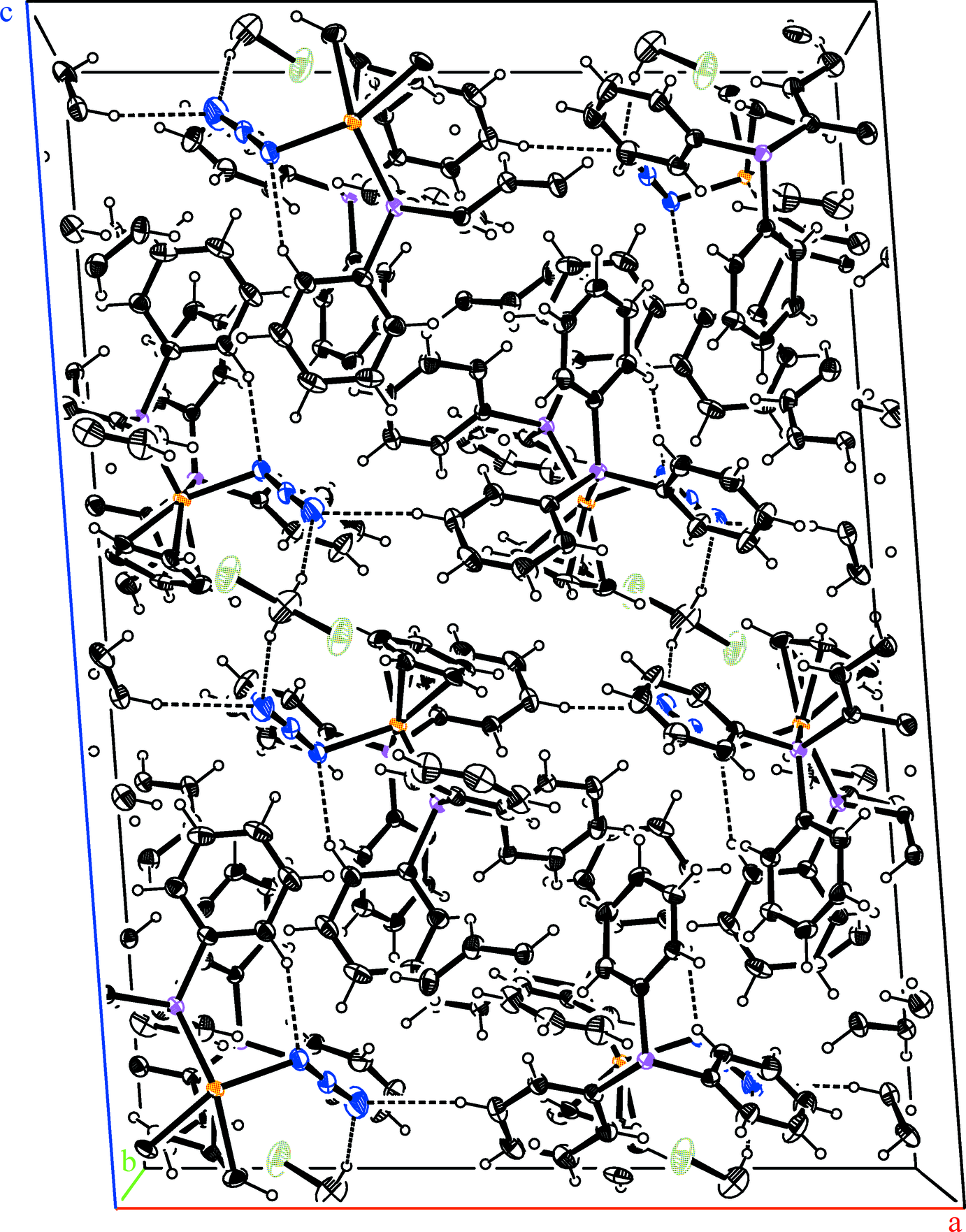

Supplement: Supplementary file 4 [file e-70-0m345-fig2.tif]
